# Duffy antigen receptor for chemokines and its involvement in patterning and control of inflammatory chemokines

**DOI:** 10.3389/fimmu.2012.00266

**Published:** 2012-08-17

**Authors:** Igor Novitzky-Basso, Antal Rot

**Affiliations:** MRC Centre for Immune Regulation, Institute of Biomedical Research, School of Infection and Immunity, University of BirminghamBirmingham, UK

**Keywords:** atypical chemokine receptors, chemokines, DARC, duffy antigen, endothelial cells, erythrocytes, inflammation, transcytosis

## Abstract

Leukocyte functions are linked to their migratory responses, which, in turn, are largely determined by the expression profile of classical chemokine receptors. Upon binding their cognate chemokines, these G-protein-coupled receptors (GPCRs) initiate signaling cascades and downstream molecular and cellular responses, including integrin activation and cell locomotion. Chemokines also bind to an alternative subset of chemokine receptors, which have serpentine structure characteristic for GPCRs but lack DRYLAIV consensus motive required for coupling to G-proteins. Duffy antigen receptor for chemokines (DARC) is a member of this atypical receptor subfamily. DARC binds a broad range of inflammatory CXC and CC chemokines and is expressed by erythrocytes, venular endothelial cells, and cerebellar neurons. Erythrocyte DARC serves as blood reservoir of cognate chemokines but also as a chemokine sink, buffering potential surges in plasma chemokine levels. Endothelial cell DARC internalizes chemokines on the basolateral cell surface resulting in subsequent transcytosis of chemokines and their immobilization on the tips of apical microvilli. These DARC-mediated endothelial cell interactions allow chemokines produced in the extravascular tissues to optimally function as arrest chemokines on the luminal endothelial cell surface.

## DUFFY BLOOD GROUP ANTIGEN

The Duffy antigen receptor for chemokines (DARC) has recently become the focus of studies investigating interactions of inflammatory chemokines with erythrocytes during systemic inflammatory responses as well as with venular endothelial cells during chemokine-induced leukocyte adhesion and emigration. These studies uncovered new functional facets of this rather “old” molecule. DARC was first described in 1950 as the Duffy blood group antigen (Cutbush and Mollison, 1950; Cutbush et al., 1950). An antibody termed anti-Fy^a^ present in the plasma of a polytransfused hemophiliac, Mr. Duffy, was found to cause a delayed hemolytic transfusion reaction. In the following year, an antibody to the antithetic antigen, Fy^b^, was found in a multigravida exposed to fetal Fy^b^ erythrocytes (Ikin et al., 1951). Subsequently, three “Duffy-positive” phenotypes were described: Fy(a+b–), Fy(a–b+), and Fy(a+b+), arising from combinations of the antithetical co-dominant *FYA* and *FYB* genes (Klein and Anstee, 2005). However, some individuals, designated “Duffy-negative,” express neither Fy^a^ nor Fy^b^ antigens, Fy(a–b–). This phenotype results from a polymorphic form of the *FYB* gene, *FYB(ES)* “erythroid silent”, present in up to 99% of West Africans and the majority of African Americans (68%; Mourant et al., 1976). The DARC (Fy) gene is located on chromosome 1, position 1922 and segregates with *Un* locus, having been the first to be assigned to an autosome in humans (Iwamoto et al., 1996). The two main alleles *FYA* and *FYB* differ in a single base substitution (125 G to A) in codon 42 in the NH_2_ extracellular domain, encoding glycine in Fy^a^ and aspartic acid in Fy^b^ (Chaudhuri et al., 1995; Iwamoto et al., 1995). The *FYB(ES)* allele has a single T to C substitution at nucleotide –*67* within the erythroid GATA-1 promoter region, 33 bp upstream from the erythroid transcription starting point and 46 bp upstream from the start of the major transcript translation codon, thus preventing DARC transcription in erythroid cells only (Tournamille et al., 1995).

Hence *FYB(ES)* Fy(a–b–) individuals still express DARC at non-erythroid sites, e.g., on endothelial cells and possibly other cells (Peiper et al., 1995; Chaudhuri et al., 1997; Horuk et al., 1997). The Duffy-negative phenotype was first linked with resistance to malaria when Fy(a–b–) volunteers exposed to the bites of *Plasmodium vivax*-infected mosquitoes, in contrast to Duffy-positives, did not develop malaria (Miller et al., 1976). This confirmed the long standing clinical observation that African populations appeared resistant to this form of malaria, noted also during the treatment of neurosyphilis by therapeutic *P. vivax* inoculation (O’Leary, 1927; Boyd and Stratman-Thomas, 1933). Further work showed that this parasite requires DARC binding for entry into the erythrocytes (Miller et al., 1975; Horuk et al., 1993a), leading to the hypothesis that the Fy(a–b–) phenotype evolved as a result of selective pressure to protect its carriers from *vivax* but not *falciparum* malaria. Geostatistical maps show that in West Africa the areas of prevalence of the Fy(a–b–) phenotype of almost 100% (Howes et al., 2011), overlap with areas of expected but absent *p. vivax* infection (Guerra et al., 2010). However, this resistance is not complete and some Fy(a–b–) populations, for example in Madagascar, both carry parasites asymptomatically and experience symptomatic *vivax* malaria (Ménard et al., 2010).

Other rare DARC polymorphisms include a C*265*T mutation in *FYB* leading to *FYX* allele and 90% reduction of DARC expression, the so called “Fy^b^ weak” phenotype, and the G*298*A polymorphism resulting in the Ala100Thr substitution (Olsson et al., 1998; Tournamille et al., 1998).

## STRUCTURAL CHARACTERISTICS OF DARC

Human DARC contains 336 amino acids (molecular weight 35733) and was first predicted to have nine trans-membrane domains (Chaudhuri et al., 1993), but later shown to have seven, akin to other chemokine receptors (Neote et al., 1994). The extracellular amino-terminal domain of 65 amino acids harbors three potential *N*-glycosylation sites at residues 16, 27, and 33 (Czerwinski et al., 2007), and epitopes Fy^a^, Fy^b^, and Fy6 (Tournamille et al., 2003). The Fy6 epitope is between Q19 and W26 residues, the binding site of the reticulocyte binding protein of *P. vivax.* Accordingly, monoclonal anti-Fy6 antibody inhibits the invasion of human erythrocytes by *P. vivax* (Tournamille et al., 2005).

## DUFFY ANTIGEN/RECEPTOR FOR CHEMOKINES

Duffy blood group antigen was designated DARC after it was shown to mediate the promiscuous binding of inflammatory CC and CXC chemokines to erythrocytes (Horuk et al., 1993b; Tournamille et al., 1997; Lee et al., 2003a; Pruenster and Rot, 2006; Ulvmar et al., 2011). DARC’s extracellular N-terminal domain, which bears the blood group determinants, is linked with the fourth extracellular domain via a disulphide bond. These domains together create an active chemokine-binding pocket (Tournamille et al., 1997, 2003). Given the absence of a DRYLAIV motif, which is required G-protein coupling, no detectable chemokine-induced cell signaling has been recorded in either the form of calcium flux (Neote et al., 1994), GTPase activity (Horuk et al., 1993b), or gene transcription (Lee et al., 2003a). Thus, DARC is classified as an atypical chemokine receptor (Nibbs et al., 2003; Pruenster and Rot, 2006; Ulvmar et al., 2011; Graham et al., 2012). However, some intracellular responses take place following DARC ligation by cognate chemokines. It was demonstrated in heterologous transfectants that chemokine binding induces redistribution of DARC from the basolateral cell membrane, via an intracellular vesicular compartment onto the apical membrane and that chemokine cargo is translocated together with DARC (Pruenster et al., 2009). Such chemokine *in situ* binding mirroring exactly the ligand specificity of DARC (Hub and Rot, 1998) as well as chemokine transcytosis and luminal surface presentation (Middleton et al., 1997) have been shown to place in venular endothelial cells *in vivo* and *ex vivo* in intact viable tissues. Unlike other atypical chemokine receptors, D6 in particular, no degradation of chemokines occurs after their internalization by DARC. Accordingly, neutrophil and monocyte migration toward cognate chemokines was enhanced across cellular monolayers expressing DARC (Lee et al., 2003a; Pruenster et al., 2009). Also i*n vivo*, chemokine injections into transgenic mice, which over-express DARC on the endothelium, induced significantly greater leukocyte recruitment (Pruenster et al., 2009). Thus endothelial DARC mediates abluminal internalization and transcellular transport of chemokines. This activity of DARC prevents the escape of soluble tissue-derived chemokine molecules into circulation and allows them to associate with the tips of luminal microvilli and stimulate firm adhesion of leukocytes. Inflammation can further up-regulate DARC expression in postcapillary venules and veins, and induce DARC to appear in vascular segments usually devoid of it (Liu et al., 1999; Segerer et al., 2000; Patterson et al., 2002; Lee et al., 2003b; Bruhl et al., 2005; Gardner et al., 2006; Geleff et al., 2010). It is not clear whether DARC over-expression is the consequence of the development of the inflammatory lesions or their pre-requisite. Primary lymphatic vessels do not express DARC although a small segment, the podoplanin-dull pre-collectors, do express DARC, suggesting that chemokines mediated cell migration may occur at this site (Wick et al., 2008).

Despite the fact that chemokine internalization by DARC does not lead to their degradation, DARC may physically remove chemokines from extracellular environments and thus, e.g., negatively influence angiogenesis induced by extravascular pro-inflammatory chemokines. This was shown in mice over-expressing endothelial DARC, which have reduced angiogenic responses to CXCL2 (Du et al., 2002) and in the context of tumor angiogenesis (Horton et al., 2007). Also, DARC-deficient mice used in a transgenic model of prostate cancer developed tumors with increased vessel density, greater intratumor angiogenic chemokine levels, and augmented growth (Shen et al., 2006). CD82, a tetraspanin which was identified as a prostate cancer metastasis suppressor gene, apparently directly interacts with DARC which thus can inhibit tumor cell proliferation and induce senescence (Bandyopadhyay et al., 2006). It appears therefore that DARC may negatively affect tumor development and metastatic spread either directly by binding to CD82 or by removing angiogenic chemokines from perivascular spaces. Additionally, DARC has been shown to heterodimerize with a classical chemokine receptor CCR5, and through this interaction down-modulate CCR5 mediated signaling responses (Chakera et al., 2008).

## THE ROLE OF DARC IN CHEMOKINE HOMEOSTASIS

Erythrocyte DARC was originally described as a chemokine “sink” (Darbonne et al., 1991) and this function was further supported when DARC was shown to reduce the levels of circulating inflammatory chemokines, thus dampening systemic leukocyte activation (Dawson et al., 2000). Chemokines in circulation can induce the desensitization of their cognate receptors. By protecting circulating leukocytes from chemokine excess, DARC may preserve subsequent leukocyte responsiveness to chemokine signals present on the endothelial surface or in the tissues. Conversely, systemic pre-exposure to chemokines may prime leukocytes for enhanced chemokine-induced migration (Brandt et al., 1998) or other effector functions (Green et al., 1996; Hauser et al., 1999). These two opposing potential outcomes may explain the following apparently conflicting observations in DARC-deficient mice exposed to various inflammatory stimuli (Dawson et al., 2000; Reutershan et al., 2009; Vielhauer et al., 2009; Mei et al., 2010; Zarbock et al., 2010). In an initial study DARC knockout (KO) animals received systemic LPS and responded by a marked increase in neutrophil infiltrate in the lungs and livers as compared to the wild type controls (Dawson et al., 2000). Another study showed that DARC KO mice have significantly less leukocyte infiltrate in the bronchoalveolar lavage in response to chemokine instilled into pulmonary airspace (Lee et al., 2003a). These experiments used DARC KOs lacking this receptor on all cells. Subsequently, bone marrow chimeras were constructed allowing the examination of respective roles of DARC on erythrocytes and endothelium (Lee et al., 2006). Here, mice lacking erythrocyte DARC had significantly fewer airspace neutrophils following intratracheal LPS instillation, suggesting that erythrocyte DARC supports leukocyte emigration. The lack of DARC in the lungs was associated with higher chemokine concentrations in the airspaces compared with mice lacking DARC on erythrocytes. In a model of LPS-inhalation-induced acute lung injury neutrophil migration into the alveolar spaces was increased in DARC KO animals, along with elevated levels of CXC chemokines (Reutershan et al., 2009). In chimeric animals, the absence of erythrocyte DARC was the most significant factor determining leukocyte trafficking. Difference between the outcomes in these two studies may be due to the divergent dose of LPS administered. With higher LPS concentrations the role for erythroid DARC as a sink may become more significant (Reutershan et al., 2009). Of note is that during severe systemic inflammation erythrocyte-bound chemokines amounted to 30% of plasma chemokine concentrations, suggesting only a limited sink effect of erythrocyte DARC during severe inflammation (Reutershan et al., 2009). Conversely, in humans, following endotoxin challenge several hundred fold increases in chemokine levels in erythrocyte lysates were noted (Mayr et al., 2008). Further investigation into the role of DARC in acute lung inflammation revealed that a lack of DARC in mice results in down-regulation of CXCR2 on neutrophils because of high levels of circulating chemokines during severe inflammation (Zarbock et al., 2010). It this study DARC was essential for chemokine-mediated leukocyte recruitment, whereby DARC KO animals were protected from acid-induced lung injury and experienced preserved oxygenation. This occurred as a result of impaired leukocyte arrest on endothelial cells and consequently reduced pulmonary neutrophil recruitment. Adoptive transfer of neutrophils showed that the latter effect is dependent on neutrophils and independent of endothelial cells and erythrocytes, suggesting that the contribution of DARC is in the maintenance of receptor expression in the environments with excess ligands. Because neutrophils, which are activated by chemokines in the systemic circulation (Colditz et al., 2007), may be passively trapped in the lung circulation and contribute to the lung damage (Rot, 1991), inflammatory models in other organs may be more revealing in dissecting local vs. systemic effects of DARC on chemokine-driven leukocyte emigration. Renal models of inflammation have shown that DARC-deficient mice are protected from ischemic and LPS-induced acute renal injury (Zarbock et al., 2007). Furthermore, chemokine presentation on renal endothelial cells was absent, and renal neutrophil recruitment was impaired, in the context of lower inflammatory chemokine levels during systemic inflammation (Zarbock et al., 2007). In contrast, Vielhauer et al. (2009) studied tubule-interstitial inflammation and glomerulonephritis in DARC-deficient mice and demonstrated that in these models macrophage and T lymphocytes were recruited equally well in DARC KO and wild type mice.

Both human and murine studies suggest that DARC can sustain inflammatory chemokines levels on erythrocytes and in plasma (Jilma-Stohlawetz et al., 2001; Fukuma et al., 2003), but the biological purpose of this reservoir function is not clear. Basal plasma CCL2 levels are one-third lower in DARC KO mice than in control wild type animals (Fukuma et al., 2003). When CCL11 or hCXCL1 were administered intravenously, these chemokines disappeared more rapidly from the plasma of DARC KOs (Fukuma et al., 2003). Duffy-negative humans were noted to have significantly lower basal CCL2 levels than Duffy-positives (Jilma-Stohlawetz et al., 2001) and following endotoxin administration, higher levels of plasma CCL2 were observed in Duffy-positive individuals as compared to the Duffy-negative ones (Mayr et al., 2008). Also CCL2 and CXCL1 levels, but not CXCL8 or CCL4 levels were higher in erythrocyte lysates of Duffy-positive individuals at baseline, whereas following endotoxin administration CCL2 and CXCL8, but not CCL4, levels increased significantly in erythrocyte lysates of Duffy-positive subjects. Given that chemokine plasma levels, including of CXCL8 (Wong et al., 2008) and CCL2 (Bozza et al., 2007) have been shown to be predictive of survival and correlate with sepsis severity, it is tempting to speculate that the loss of DARC expression may affect the outcome in sepsis. It has been recently suggested that chemokines with different binding affinities for DARC can modify the levels of other erythrocyte-bound and free plasma chemokines, affecting resultant leukocyte responses (Mei et al., 2010). In addition, heparin and activated coagulation factors can elute chemokines off erythrocyte DARC (Schnabel et al., 2010). Thus chemokines with range of affinities for DARC and other factors may significantly interfere with the ability of DARC to bind any particular chemokine introducing further complexity into mechanistic understanding of erythrocyte DARC function.

Recently, differences in plasma and serum chemokine levels were reported in persons with DARC Fy^a^ and Fy^b^ (Schnabel et al., 2010), although the mechanism for this is not apparent. Further work revealed that Fy^b^ erythrocytes have reduced surface DARC expression as compared to Fy^a^ erythrocytes; however, the binding affinity of DARC for chemokines was not appreciably different between these two phenotypes (Xiong et al., 2011). As discussed above, endothelial cells of post-capillary and collective venules and small veins express DARC, which functions here as a transcytosis receptor transporting chemokines from the basolateral to the apical side (Pruenster et al., 2009) where they are immobilized on the luminal surface. It is attractive to speculate that that individuals of alternative Fy^a^ vs. Fy^b^ DARC phenotypes may also show differences in chemokine-binding specificity and patterning by the endothelium, though to date there is no data to support this notion.

## CONCLUSION

Since the discovery of its chemokine-binding properties, DARC has been mainly considered as a “decoy” receptor scavenging its ligands. Recent research shed new light on much more multifaceted activities of DARC. On erythrocytes, DARC acts on the one hand as a blood chemokine sink and, on the other, as a reservoir of cognate chemokines buffering the bursts in their blood levels, and maintaining these, respectively. Both of these functions are absent in individuals with FYB(ES) DARC “negative” polymorphism. Future work should uncover molecular and cellular mechanisms explaining how the lack of erythrocyte chemokine sink and depot functions in these DARC-negative individuals affects pathomechanisms in various inflammatory diseases and cancer. In endothelial cells DARC functions as a transcytosis receptor leading to correct patterning of chemokines on the tissue–blood interface in venules and veins, thus supporting optimal chemokine-induced leukocyte endothelial cell adhesion and subsequent leukocyte emigration.

## Conflict of Interest Statement

The authors declare that the research was conducted in the absence of any commercial or financial relationships that could be construed as a potential conflict of interest.
